# LMKB/MARF1 Localizes to mRNA Processing Bodies, Interacts with Ge-1, and Regulates *IFI44L* Gene Expression

**DOI:** 10.1371/journal.pone.0094784

**Published:** 2014-04-22

**Authors:** Donald B. Bloch, Pingcheng Li, Emily G. Bloch, Daniel F. Berenson, Rita L. Galdos, Pankaj Arora, Rajeev Malhotra, Connie Wu, Weihong Yang

**Affiliations:** 1 Center for Immunology and Inflammatory Diseases, Department of Medicine, Massachusetts General Hospital and Harvard Medical School, Boston, Massachusetts, United States of America; 2 Anesthesia Center for Critical Care Research, Department of Anesthesia, Critical Care and Pain Medicine, Massachusetts General Hospital and Harvard Medical School, Boston, Massachusetts, United States of America; 3 Center for Human Genetic Research, Massachusetts General Hospital, Harvard Medical School, Boston, Massachusetts, United States of America; 4 Cardiovascular Research Center, Cardiology Division of the Department of Medicine, Massachusetts General Hospital and Harvard Medical School, Boston, Massachusetts, United States of America; Korea University, Republic of Korea

## Abstract

The mRNA processing body (P-body) is a cellular structure that regulates the stability of cytoplasmic mRNA. MARF1 is a murine oocyte RNA-binding protein that is associated with maintenance of mRNA homeostasis and genomic stability. In this study, autoantibodies were used to identify Limkain B (LMKB), the human orthologue of MARF1, as a P-body component. Indirect immunofluorescence demonstrated that Ge-1 (a central component of the mammalian core-decapping complex) co-localized with LMKB in P-bodies. Two-hybrid and co-immunoprecipitation assays were used to demonstrate interaction between Ge-1 and LMKB. The C-terminal 120 amino acids of LMKB mediated interaction with Ge-1 and the N-terminal 1094 amino acids of Ge-1 were required for interaction with LMKB. LMKB is the first protein identified to date that interacts with this portion of Ge-1. LMKB was expressed in human B and T lymphocyte cell lines; depletion of LMKB increased expression of *IFI44L*, a gene that has been implicated in the cellular response to Type I interferons. The interaction between LMKB/MARF1, a protein that contains RNA-binding domains, and Ge-1, which interacts with core-decapping proteins, suggests that LMKB has a role in the regulation of mRNA stability. LMKB appears to have different functions in different cell types: maintenance of genomic stability in developing oocytes and possible dampening of the inflammatory response in B and T cells.

## Introduction

Components of the cytoplasmic mRNA processing body (P-body) have important roles in many aspects of mRNA metabolism. P-body components participate in the 5′->3′ degradation of mRNA and in microRNA-mediated post-transcriptional gene silencing (reviewed in [1], [2], [3], [4]). P-body components also have a role in the transmission of messages in neurons [5], [6], [7], the cellular response to viral infections [8], [9], the regulation of genomic stability [10], and in the earliest stages of embryonic development [11], [12]. In view of the contribution of P-bodies to this broad spectrum of cellular functions, and the relatively limited number of P-body proteins that have been identified so far, it seems likely that the full complement of P-body components has not yet been defined.

Human Limkain B (LMKB, also known as LKAP, KIAA0430) was originally identified as a protein that interacts with Lim-kinase 1 in a yeast two-hybrid assay (http://www.ncbi.nlm.nih.gov/nucleotide/18146749). LMKB is a member of a family of proteins that are predicted to bind RNA. Su and colleagues observed that a homozygous mutation in the murine orthologue of LMKB, MARF1 (Meiosis Arrest Female 1), resulted in defective oogenesis and impaired regulation of retrotransposon activity [13], [14].

Approximately 5% of patients with primary biliary cirrhosis (PBC) produce antibodies directed against P-bodies [15]. PBC is an autoimmune disease of unknown etiology characterized by the gradual destruction of intrahepatic, medium-sized biliary ductules leading to liver failure (reviewed in [16]). The humoral autoimmune response in PBC patients may begin years, if not decades, before the onset of overt disease [17]. During this interval, patients may produce high-titer and high-affinity antibodies directed against cellular structures, and the antibody response may spread from one to many proteins in a macromolecular complex. In previous studies, Eystathioy and colleagues used autoantibodies to identify P-body component GW182 [18] and we used PBC sera to identify P-body proteins Ge-1 (also known as HEDLS and EDC4), RAP55 and YB1 [19], [20], [21]. In a continuation of these studies, we used patient serum to screen a proteomic array and identified Limkain B (LMKB) as a novel P-body component.

## Materials and Methods

### Ethics Statement

After written informed consent was obtained, blood samples were obtained from patients with autoantibodies directed against mRNA processing bodies. This study was reviewed and approved by the Partners Health Care Human Institutional Review Board.

### Plasmids and Antisera

A cDNA corresponding to the LMKB sequence NM_014647 was ligated into pEGFP (Clontech, Palo Alto, CA) and pDsRFP (Invitrogen, Carlsbad, CA) to produce plasmids encoding GFP-LMKB and RFP-LMKB respectively. DNA fragments encoding LMKB amino acids 1457–1742, 1622–1742, 1–687, 389–1199, and 1–1642 were prepared using restriction enzymes or oligonucleotides and PCR and the fragments were ligated into pEGFP. A eukaryotic expression plasmid encoding DCP1 was provided by J. Lykke-Andersen (University of California, San Diego). A plasmid encoding full-length Ge-1 fused to GFP and an exogenous nuclear localization sequence (NLS) corresponding to the SV40 T antigen NLS (PKKKRKV) was previously reported [22]. Plasmids encoding GFP-NLS-Ge-1(935–1437), GFP-NLS-Ge-1(630–1437), GFP-Ge-1(1–1094), GFP-Ge-1(1–935), and GFP-Ge-1(104–1094) were prepared using restriction enzymes or oligonucleotides and PCR. The nucleotide sequence of all PCR products was confirmed. The successful production of each protein fragment was confirmed by immunoblot.

Serum from a patient with PBC (patient 0081) was identified in a study to determine the clinical significance of autoantibodies in PBC [23]. A second human serum (from patient 0121), containing antibodies directed against Ge-1, was previously described [21]. Serum 0121 did not contain anti-LMKB antibodies, as determined by immunoblot (not shown) and by lack of reactivity with LMKB on the protein macroarray. Mouse anti-GFP antibodies (Invitrogen), rabbit anti-GFP antibodies (Santa Cruz Biotechnology), mouse anti-FLAG antibodies (Sigma-Aldrich) and rabbit anti-RFP antiserum (Clontech) were obtained from the respective companies. Antibodies directed against LMKB amino acids 1457–1742 were prepared at Cocalico Biologics, following a standard protocol. Species-specific secondary antisera, conjugated to FITC, rhodamine, or coumarin were obtained from Jackson ImmunoResearch Laboratories.

### Cell Culture and Immunohistochemistry

Hut78, HL60, HEp-2, and COS-7 cell lines were purchased from American Type Cell Collection. BJAB and NB4 cell lines were obtained from E. Kieff (Brigham and Women’s Hospital, Boston) and M. Lanotte (Inserm U-496, Paris), respectively. Neuroblastoma cell lines LA1N and LA1S were provided by S. Cohn (University of Chicago Medical School). Hut78, BJAB, NB4 and HL60 cells were cultured in RPMI; HEp-2, COS-7, LA1N and LA1S cell lines were maintained in DMEM. RPMI and DMEM were supplemented with 10% fetal calf serum, L-glutamine (2 mM), penicillin (200 U/ml) and streptomycin (200 µg/ml). To induce stress granule formation in Hut78 cells, the cells were exposed to sodium arsenite (0.5 mM) for 1 hour at 37°C. Prior to immunofluorescent staining, non-adherent cells (Hut78 and BJAB) were subjected to cytocentrifugation at 500 rpm for 5 minutes. Adherent cells (HEp-2) were grown in tissue culture chambers (Nunc Inc, Naperville, Ill). Both adherent and non-adherent cells were subsequently fixed in 4% paraformaldehyde in PBS and permeabilized with 100% methanol. Cells were stained with primary and secondary antisera as previously described [15]. Slides were examined using a Nikon Eclipse 80i UV microscope.

### Preparation of GST Fusion Protein, SDS-PAGE and Immunoblotting

Plasmids encoding GST or GST fused to LMKB(1457–1742) were used to produce recombinant protein in *E.coli*. Protein extracts were fractionated in SDS-10% polyacrylamide gels and transferred to a PVDF membrane. The membrane was incubated in blocking solution (PBS containing 5% nonfat milk) for 1 hour at room temperature, followed by human serum 0081 diluted 1∶5,000 in blocking solution. Bound human antibodies were detected using HRP-conjugated goat anti-human IgG antiserum (Jackson ImmunoResearch) and chemiluminescence.

### Co-immunoprecipitation Assay

A co-immunoprecipitation assay was used to detect interaction between carboxyl terminal portions of LMKB and Ge-1. COS-7 cells were transfected with combinations of plasmids encoding GFP, GFP-LMKB(1457–1742), GFP-LMKB(1622–1742), FLAG-Ge-1 and FLAG alone (as indicated) using the Neon transfection system (Life Technologies, Grand Island, NY). Extracts were prepared from transfected COS-7 cells and incubated with mouse anti-GFP antibodies and protein G conjugated to Sepharose beads. Adherent proteins were eluted by boiling in SDS-gel sample buffer, fractionated by PAGE, and transferred to a PVDF membrane. Co-precipitated FLAG-Ge-1 was detected using anti-FLAG antibodies. GFP, GFP-LMKB(1457–1742) and GFP-LMKB(1622–1742) were detected using rabbit anti-GFP antibodies.

### Screening the High-density Protein Macroarray

A high-density protein macroarray prepared from phytohemagglutinin-treated human T-cells was obtained from Imagenes (formerly RZPD). The membrane was rinsed with 100% ethanol at room temperature and incubated with blocking solution as previously described [21]. The membrane was incubated overnight at 4°C with serum 0081 diluted 1∶5,000 in blocking solution. Bound antibodies were detected using HRP-conjugated rabbit anti-human IgG antiserum (Sigma-Aldridge) and chemiluminescence.

### Modified Two-hybrid Assay

A previously described modified two-hybrid assay was used to detect interactions between LMKB and Ge-1 inside HEp-2 cells [22]. For these studies, plasmids were transfected into HEp-2 cells using the Effectene transfection system. Cells were fixed and stained 24 hours after transfection.

### Inhibition of LMKB Expression Using Small Interfering RNA (siRNA)

*LMKB* mRNA knockdown was achieved using siRNA contained within lentivirus transduction particles (Sigma-Aldrich catalogue# TRCN0000061918). A lentivirus containing siRNA directed against red fluorescent protein was used as a control. SiRNAs were transduced into BJAB (B lymphocyte) and Hut78 (T lymphocyte) cell lines as directed by the manufacturer, using a multiplicity of infection of 10 particles per cell. The levels of *LMKB* mRNA and protein in cell lines were assessed using qRT-PCR and indirect immunofluorescence, respectively.

### Gene Expression

Total RNA was extracted from cell lines using RNeasy extraction kit (Promega). Complementary DNA was synthesized using M-MLV reverse transcription (Promega) and *LMKB*, *Line1*, *PPP2cb*, *IFI44L* transcript levels were measured by qPCR in a Mastercycler ep realplex 2 (Eppendorf) using hydrolysis probes (TaqMan Gene expression assays, Applied Biosystems). Changes in relative gene expression, normalized to 18S RNA, were determined using the relative C_T_ method.

### Microarray Analysis

Total RNA was extracted from three sets each of LMKB-depleted, and RFP siRNA-treated (control), BJAB cells. Biotin-labeled, fragmented cDNA was prepared by the Microarray Core facility at the Dana Farber Cancer Institute (Boston, MA) and used to screen Affymetrix GeneChip Human Gene 1.0 ST arrays (Affymetrix, Santa Clara, CA). Results were analyzed using the probe logarithmic intensity error (PLIER) method [24]. Values of signal intensity were log_2_ transformed and normalized before Student t test was used to perform probe-specific comparisons. Genes were considered absent if the group’s average signal intensity was below the 80^th^ percentile level of negative control values. Genes with a statistically significant (P<0.05) change of at least +/−1.8 fold were considered differentially expressed [25]. Microarray data are deposited in the Gene Expression Omnibus (www.ncbi.nlm.nih.gov/geo, dataset GSE55974).

## Results

### Autoantibodies in the Serum of Patient 0081 React with Many Known P-body Proteins and with a New P-body Component, LMKB

Using indirect immunofluorescence and the human HEp-2 cell line as substrate, we observed that antibodies in serum from PBC patient 0081 reacted with 5–20 cytoplasmic foci per cell, a characteristic P-body staining pattern. This serum reacted with previously identified P-body components Ge-1, RAP55, EDC3 and DCP1 by immunoblot (data not shown). Because serum 0081 reacted with many P-body components, it seemed likely that the serum might contain antibodies directed against as yet undiscovered P-body proteins. The serum was therefore used to screen a proteomic array prepared from phytohemagglutinin-treated human T lymphocytes. In a previous study, six PBC sera were used to identify 67 immunoreactive proteins in the array [21]. Autoantibodies in serum 0081 reacted with 48 of the previously reported proteins, as well as eight new proteins: CCDC6, TNFSF, RASA3, TRIM38, Kri1, EEF2, RBEL1 and KIAA0430 (the last also known as LMKB). Of these 8 proteins, LMKB was chosen for further investigation because a previous study suggested that LMKB is an autoantigen that localizes to a “subset of peroxisomes” [26], a staining pattern that might appear similar to the mRNA P-body pattern.

**Figure 1:**
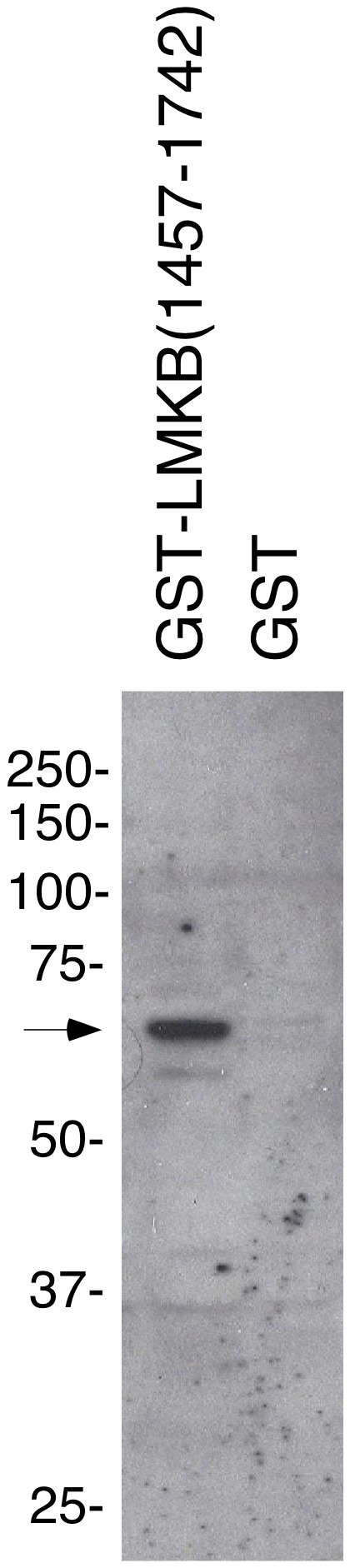
Antibodies in the serum of patient 0081 react with LMKB. The human serum contained antibodies directed against GST-LMKB amino acids 1457–1742, but did not react with GST alone. Black arrow indicates location of the GST-LMKB fusion protein.

Dunster and colleagues showed that the C-terminus of LMKB contained at least one of the autoantigen’s immunoreactive domains [26]. To confirm that LMKB was a target of antibodies in serum 0081, the serum was incubated with an immunoblot prepared using GST fused to the carboxyl terminus of LMKB (amino acids 1457–1742). Serum 0081 contained antibodies directed against this portion of the protein (Figure 1).

**Figure 2:**
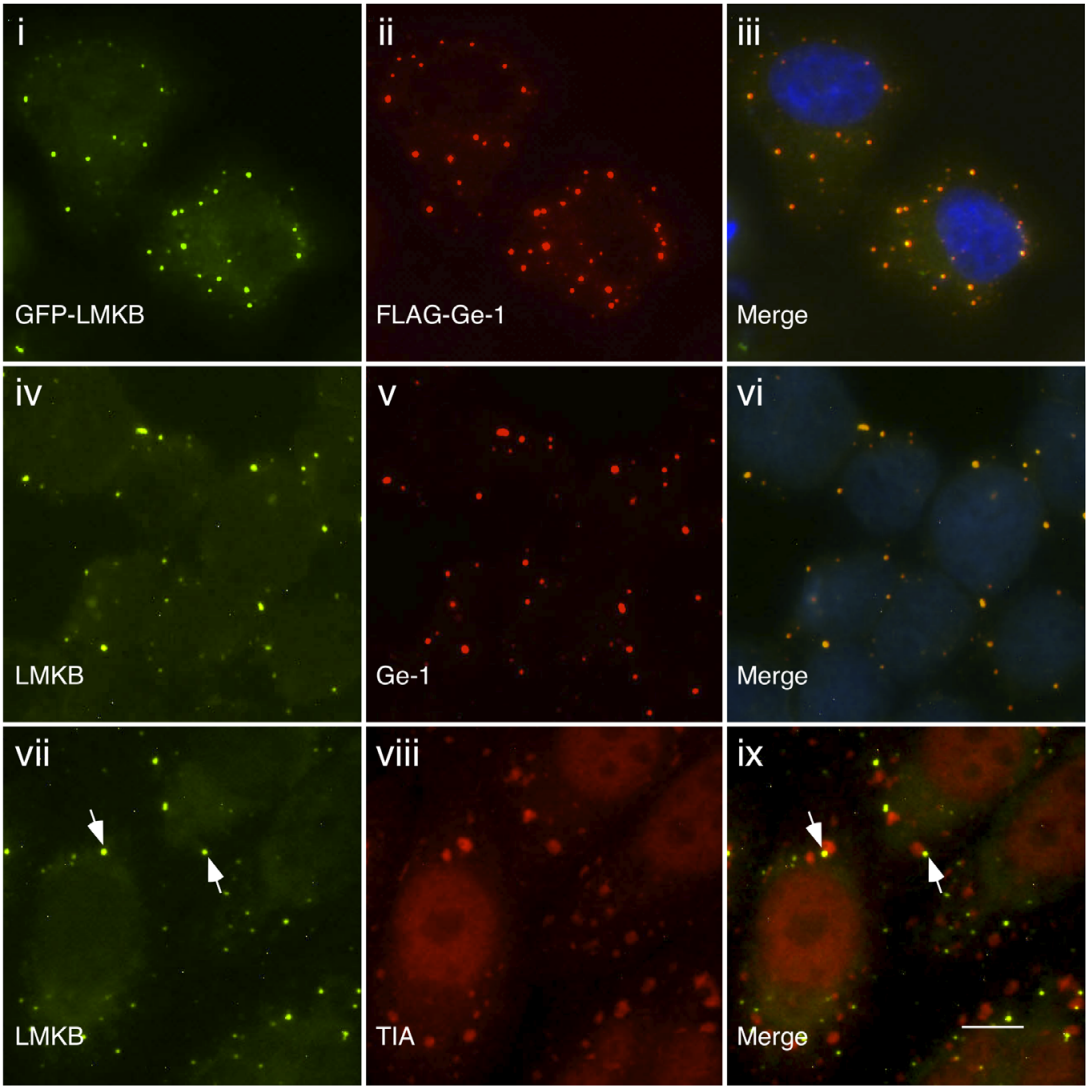
Indirect immunofluorescence shows that LMKB localizes to P-bodies. GFP-LMKB (green, i) localized to discrete, dot-like structures in the cytoplasm of transfected HEp-2 cells and co-localized with co-expressed FLAG-Ge-1 (red, ii). To determine the cellular location of *endogenous* LMKB, rabbit anti-LMKB antiserum was used to stain Hut78 cells. LMKB (green, iv) co-localized with Ge-1 (red, v), identified using human serum 0121. After exposure to arsenite for 1 hour, TIA (a marker of stress granules) was detected in cytoplasmic granules (red, viii). LMKB (green, vii) did not co-localize with TIA in stress granules, but was instead detected in adjacent P-bodies. Merge of fluorescence in i and ii, iv and v, vii and viii is shown in iii, vi and ix. DAPI staining in iii and vi (blue) indicates the location of nuclei. White arrows in vii and ix indicate representative LMKB-containing P-bodies adjacent to stress granules. White bar in ix indicates 5.0 µm.

To further investigate the cellular location of LMKB, a plasmid encoding full-length LMKB fused to green fluorescent protein (GFP) was prepared and co-expressed with FLAG-Ge-1 in HEp-2 cells. The two proteins co-localized in cytoplasmic dots (Figure 2, i–iii). Co-expression of GFP-LMKB and FLAG-DCP1 in HEp-2 cells also resulted in co-localization of the two proteins in cytoplasmic dots (Figure S1, i–iii). These results confirm that LMKB is a component of mRNA P-bodies.

### The Structure of LMKB and Expression of LMKB in Human Cell Lines

LMKB is a member of a family of proteins that contains motifs found in Oskar and vertebrate Tudor (“OST”) proteins [27]. These motifs are predicted to adopt a winged helix-turn-helix (“HTH”) fold that is capable of binding double-stranded RNA. LMKB contains eight C-terminal OST motifs between amino acids 879–1557 (http://www.ncbi.nlm.nih.gov/Structure/cdd/wrpsb.cgi?SEQUENCE=124487213&FULL, [28]). LMKB has two additional RNA recognition motif (RRM)-type RNA binding domains, between amino acids 510–600 and 792–867. The N-terminus of LMKB (amino acids 351–493) contains a predicted globular structure that may serve as a cation binding domain (http://www.ncbi.nlm.nih.gov/Structure/cdd/cddsrv.cgi?ascbin=8&maxaln=10&seltype=2&uid=199896).

Rabbit anti-LMKB antibodies were used to probe the expression of LMKB in a panel of human cell lines. Because we originally identified LMKB in a protein array derived from T lymphocytes, we stained the human helper T cell line Hut78 for the protein. In these cells, LMKB localized to cytoplasmic dots and co-localized with endogenous Ge-1 (Figure 2, iv-vi). LMKB was also detected in P-bodies in the human B cell line BJAB (not shown). LMKB was not detected by indirect immunofluorescence in EBV-transformed B cells lines, in myeloid cell lines HL60 and NB4, in neuroblastoma cell lines LA1N and LA1S, or in HEp-2 cells.

To examine the effect of stress on the cellular distribution of LMKB, Hut78 cells were treated with arsenite for 1 hour and then zstained with antibodies directed against LMKB and the stress granule marker TIA. LMKB-containing P-bodies localized adjacent to stress granules, but LMKB was not detected within stress granules (Figure 2, vii-ix). LMKB is therefore similar to Ge-1 in that it does not localize to stress granules [29]. In contrast, other P-body components, such as RAP55, RCK, Eif4E, Xrn1 and YB-1 were previously found to localize to stress granules in response to arsenite treatment [20], [30], [31].

### LMKB Interacts with Ge-1

**Figure 3:**
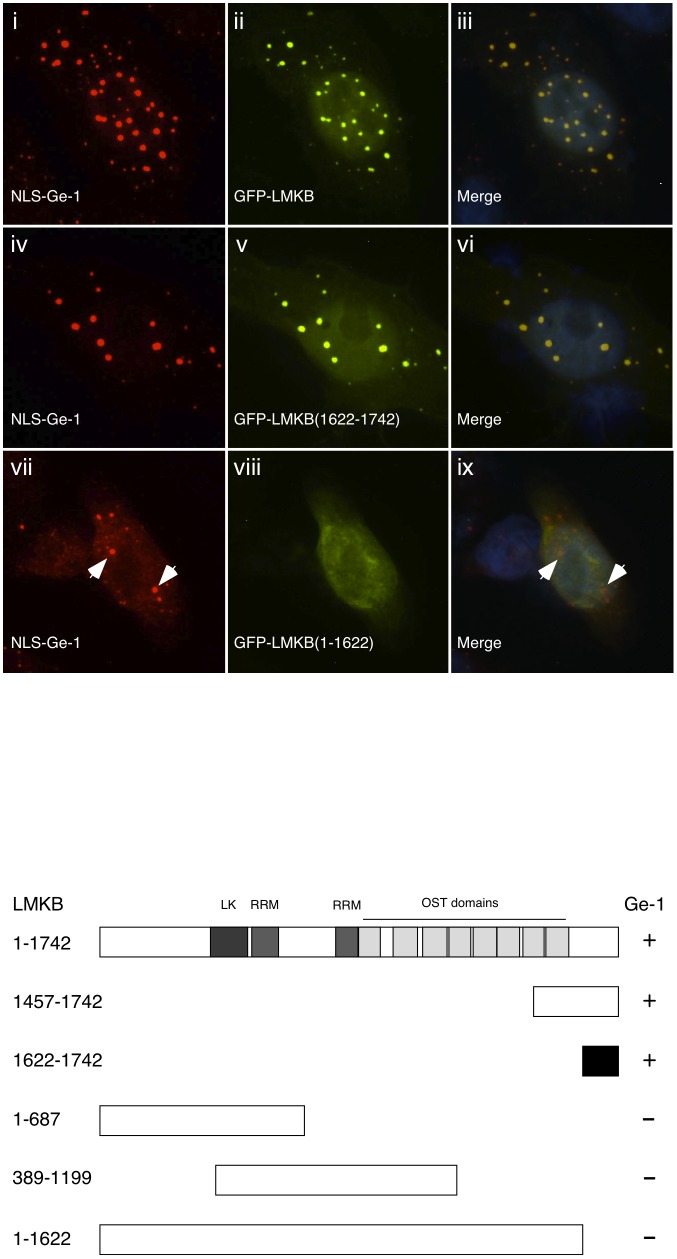
A. A modified two-hybrid assay was used to test for interaction between LMKB and Ge-1 and to identify the portion of LMKB that mediates interaction with Ge-1. Expression of a plasmid encoding Ge-1 fused to a nuclear localization sequence (NLS) shifted some of the protein from the cytoplasm to the nucleus of HEp-2 cells (red, i, iv and vii), where it localized to dot-like structures. Co-transfection of plasmids encoding GFP fused to full-length LMKB (green, ii) and NLS-Ge-1 resulted in co-localization of the two proteins in nuclear dots. GFP-LMKB fragment fusion proteins encoding amino acids 1622–1742 (green, v) also co-localized with co-expressed NLS-Ge-1 (red, iv) in nuclear dots. GFP-LMKB fragments encoding 1–687 and 389–1199 (both not shown) and 1–1622 (green, viii) did not co-localize with NLS-Ge-1 in nuclear dots. Merge of fluorescence in i and ii, iv and v, vii and viii is shown in iii, vi and ix. DAPI staining (blue) in iii, vi and ix indicates the location of nuclei. Human serum 0121 containing anti-Ge-1 antibodies and mouse monoclonal anti-GFP antibodies were used to detect Ge-1 and GFP, respectively. White arrows in vii and ix indicate nuclear dots containing NLS-Ge-1. White bar in ix indicates 5.0 µm. **B**. Schematic representation of the structure of LMKB and summary of results. LMKB contains an N-terminal globular domain (“LK”, amino acids 351–493) that may serve as a cation binding domain. Two RRM-type RNA binding domains are located between amino acids 510–600 and 792–867. The C-terminus of LMKB contains eight helix-turn-helix folds (“OST domains”) that are predicted to bind to dsRNA (amino acids 879–937, 1004–1074, 1100–1170, 1176–1247, 1259–1330, 1337–1406, 1412–1484, 1487–1557; numbering system is as indicated in GenBank #NM_014647). + indicates an interaction between a LMKB fragment and Ge-1; - indicates a fragment of LMKB that does not interact with Ge-1. The black rectangle indicates the smallest tested portion of LMKB that interacts with Ge-1.

Because LMKB co-localized with Ge-1 in P-bodies and had a similar cellular distribution after exposure to arsenite, we tested the possibility that LMKB interacts with Ge-1. The large size of both Ge-1 and LMKB, and the relative instability of full-length LMKB, made it technically difficult to demonstrate interaction using co-immunoprecipitation. We therefore used a modified two-hybrid assay to test for interaction between the two proteins inside cells. As previously described [22], this assay takes advantage of the observation that Ge-1, when fused to an exogenous nuclear localization sequence (NLS) and expressed in HEp-2 cells, localizes to nuclear dots. Co-expression of NLS-Ge-1 with proteins that interact with Ge-1, including DCP1, DCP2 or Ge-1 itself, resulted in localization of both proteins to these nuclear structures. In contrast, proteins that do not interact with Ge-1, including RCK, EDC3 and RAP55, did not co-localize with NLS-Ge-1 [22]. After co-expression of NLS-Ge-1 and GFP-LMKB in HEp-2 cells, both NLS-Ge-1 and GFP-LMKB were detected in nuclear dots (Figure 3A, i–iii). When expressed without NLS-Ge-1, GFP-LMKB localized to the cytoplasm (see Figure 2i). The results suggest that LMKB and Ge-1 are able to interact within HEp-2 cells.

To identify the portion of LMKB that mediates interaction with Ge-1, fragments of LMKB fused to GFP were co-expressed with NLS-Ge-1 in HEp-2 cells. LMKB fragments containing the C-terminal portion of the protein, including amino acids 1457–1742 (not shown) and 1622–1742 (Figure 3A, iv–vi), were sufficient to mediate co-localization with NLS-Ge-1 in nuclear dots. N-terminal portions of LMKB, including fragments containing amino acids 1–687, 389–1199 (both not shown), and 1–1622 (Figure 3A, vii–ix) did not co-localize with NLS-Ge-1. The LMKB fragment containing amino acids 1622–1742 was the smallest portion of LMKB tested that was capable of interacting with Ge-1. The results are summarized in Figure 3B.

**Figure 4:**
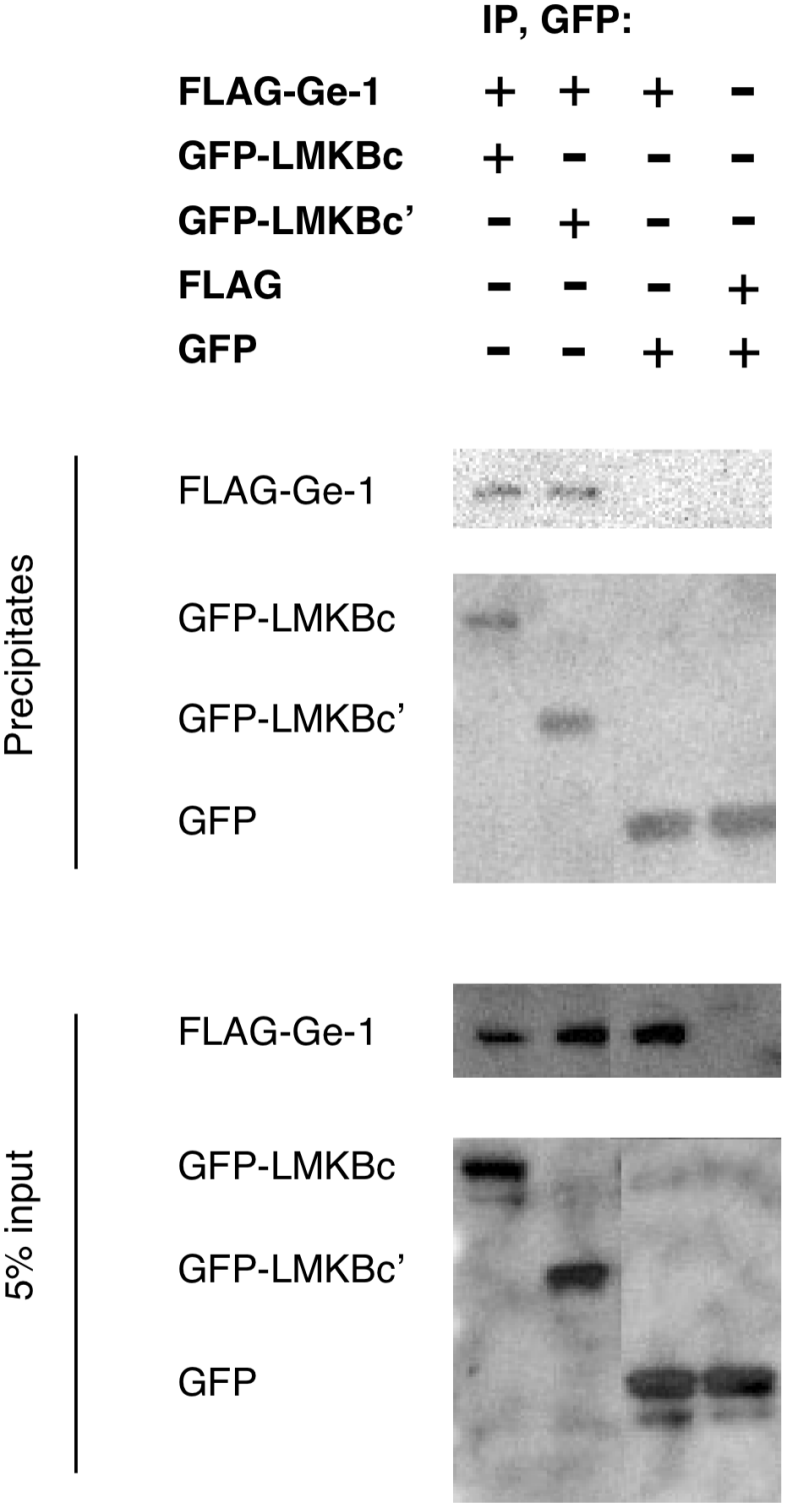
Ge-1 co-precipitates with C-terminal fragments of LMKB. COS-7 cells were transfected with plasmids encoding FLAG-Ge-1, GFP-LMKBc (containing amino acids 1457–1742), GFP-LMKBc’ (amino acids 1622–1742), FLAG and GFP as indicated. Extracts were prepared and incubated with mouse anti-GFP antibody and protein G coupled to Sepharose beads. Precipitates (top two panels) are compared with 5% of the total COS-7 cell extract input (bottom two panels). FLAG-Ge-1 co-precipitated with GFP-LMKBc(1457–1742) and with GFP-LMKBc’(1622–1742), but not with GFP alone.

To confirm that the C-terminal portion of LMKB was sufficient to mediate interaction with Ge-1, plasmids encoding GFP-LMKB(1457–1742), GFP-LMKB(1622–1742) or GFP alone were co-transfected with FLAG-Ge-1 into COS-7 cells. Mouse anti-GFP was able to co-precipitate FLAG-Ge-1 in extracts prepared from cells expressing GFP-LMKB(1457–1742) and FLAG-Ge-1, and GFP-LMKB(1622–1742) and FLAG-Ge-1, but not from cells expression GFP and FLAG-Ge-1 (Figure 4).

**Figure 5:**
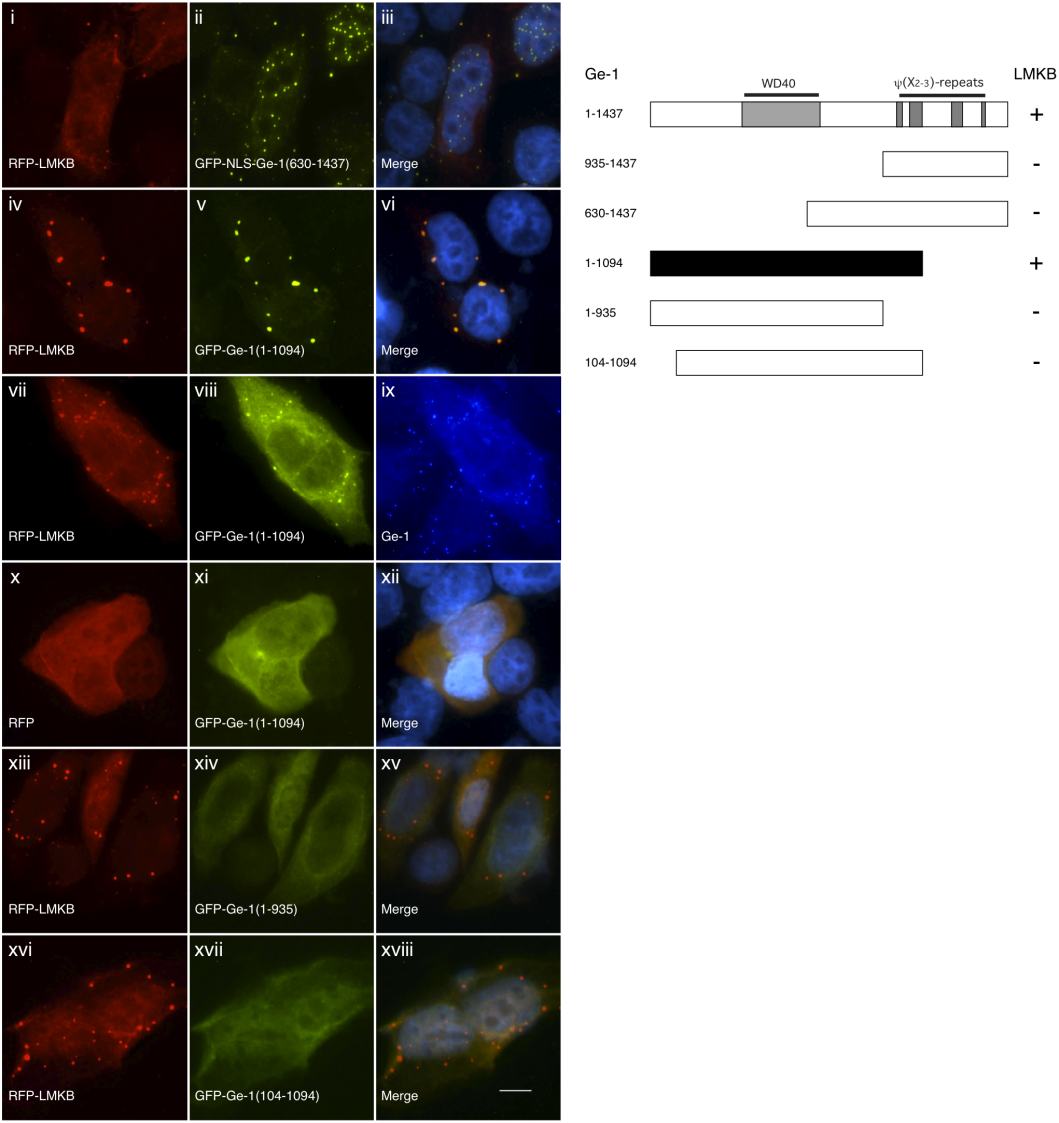
A. Delineation of the smallest portion of Ge-1 that mediates interaction with LMKB. GFP-NLS-Ge-1 fragment (amino acids 630–1437) localized to nuclear dots (green, ii) in HEp-2 cells, but RFP-LMKB remained in P-bodies and was not detected in the nucleus (red, i), suggesting that N-terminal amino acids in Ge-1 are required for interaction with LMKB. To identify the N-terminal portion of Ge-1 that interacts with LMKB, RFP-LMKB was tested for the ability to recruit N-terminal fragments of Ge-1 to P-bodies. In the presence of RFP-LMKB (red, iv), GFP-Ge-1(1–1094) localized to cytoplasmic dots resembling P-bodies (green, v). In cells expressing RFP-LMKB (red, vii) and GFP-Ge-1(1–1094) (green, viii), both proteins co-localized with endogenous Ge-1 (blue, ix), confirming that these structures are P-bodies. In the absence of LMKB (RFP alone, red, x), GFP-Ge-1(1–1094) did not localize to P-bodies (green, xi), but was instead distributed throughout the cytoplasm. Smaller fragments of Ge-1, including amino acids 1–935 (green, xiv) and 104–1094 (green, xvii) did not co-localize with co-expressed LMKB in P-bodies. Merge of fluorescence in i and ii, iv and v, x and xi, xiii and xiv, and xvi and xvii is shown in iii, vi, xii, xv and xviii. DAPI staining (blue in the merged panels) indicates the location of nuclei. White bar in xviii indicates 5.0 µm. **B.** Schematic representation of Ge-1 and summary of results. The N-terminus of Ge-1 contains a WD40 repeat domain. The C-terminus contains four regions that have repeating hydrophobic residue periodicity (ψ(X_2-3_)-repeat domains). + indicates a fragment of Ge-1 that interacts with LMKB; - indicates a fragment of Ge-1 that does not interact with LMKB. The black rectangle indicates the smallest tested portion of Ge-1 that interacts with LMKB.

To identify the portion of Ge-1 that mediates interaction with LMKB, plasmids encoding fragments of Ge-1 fused to GFP and NLS were co-expressed with a plasmid encoding red fluorescent protein (RFP) fused to LMKB. Previous studies showed that the carboxyl portion of Ge-1 was sufficient to mediate interaction with DCP1, DCP2, and Ge-1 itself [22]. However, GFP-NLS-Ge-1 fusion proteins containing amino acids 935–1437 (not shown) or 630–1437 (Figure 5A, i–iii) did not recruit RFP-LMKB into the nucleus, suggesting that motifs in the N-terminal portion of Ge-1 are required for interaction between the two proteins. Unfortunately, even when fused to an exogenous NLS, the N-terminal portion of Ge-1 did not localize to the nucleus (data not shown). In addition, as previously reported [29], the N-terminal portion of. Ge-1 did not localize to P-bodies. Therefore, to identify the N-terminal portion of Ge-1 that interacts with LMKB, the two-hybrid assay was further modified so as to test for the ability of RFP-LMKB to mediate localization of N-terminal Ge-1 fragments to P-bodies. Co-expression of RFP-LMKB and GFP-Ge-1(1–1094) resulted in both proteins localizing to cytoplasmic dots (Figure 5A, iv–vi). RFP-LMKB and GFP-Ge-1(1–1094) co-localized with endogenous Ge-1, identified using human serum containing anti-Ge-1 antibodies, suggesting that the cytoplasmic dots containing RFP-LMKB and GFP-Ge-1(1–1094) are P-bodies (Figure 5A, vii–ix). It seems likely that LMKB is able to bind more than one Ge-1 molecule and is thereby able to recruit GFP-Ge-1(1–1094) to endogenous Ge-1 in P-bodies. In cells transfected with plasmids encoding RFP (not fused to LMKB) and GFP-Ge-1(1–1094), GFP-Ge-1(1–1094) failed to localize to cytoplasmic dots, but was instead distributed throughout the cytoplasm (Figure 5A, x–xii). GFP-Ge-1 fragments lacking an additional 159 amino acids from the C-terminus, or 104 amino acids from the N-terminus, of Ge-1 did not co-localize with RFP-LMKB in P-bodies (Figure 5A, xiii–xviii). These results, which are summarized in Figure 5B, suggest that Ge-1 amino acids 1–1094 contain the portion of the protein that mediates interaction with LMKB.

### The Effects of LMKB Depletion in BJAB and Hut78 Cell Lines

**Figure 6:**
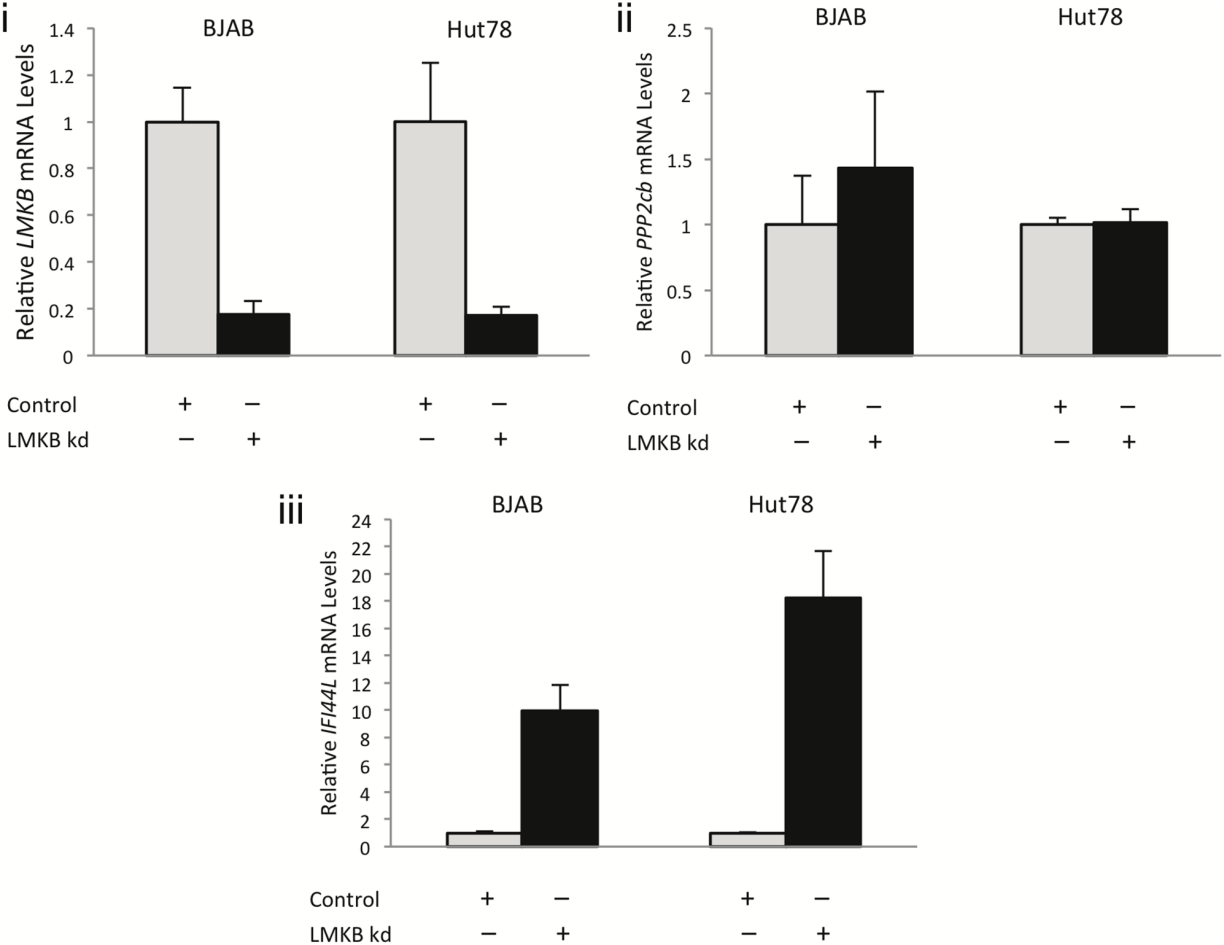
The effects of LMKB depletion on gene expression in BJAB and Hut78 cells . SiRNA-mediated knock-down of LMKB in BJAB and Hut78 cells resulted in a greater than 80% decrease in the level of *LMKB* mRNA (i). LMKB depletion had no effect on the level of *PPP2cb* mRNA in either cell line (ii). The level of *IFI44L* mRNA was increased in both BJAB and Hut78 cell lines after LMKB knock-down (iii).

**Figure 7:**
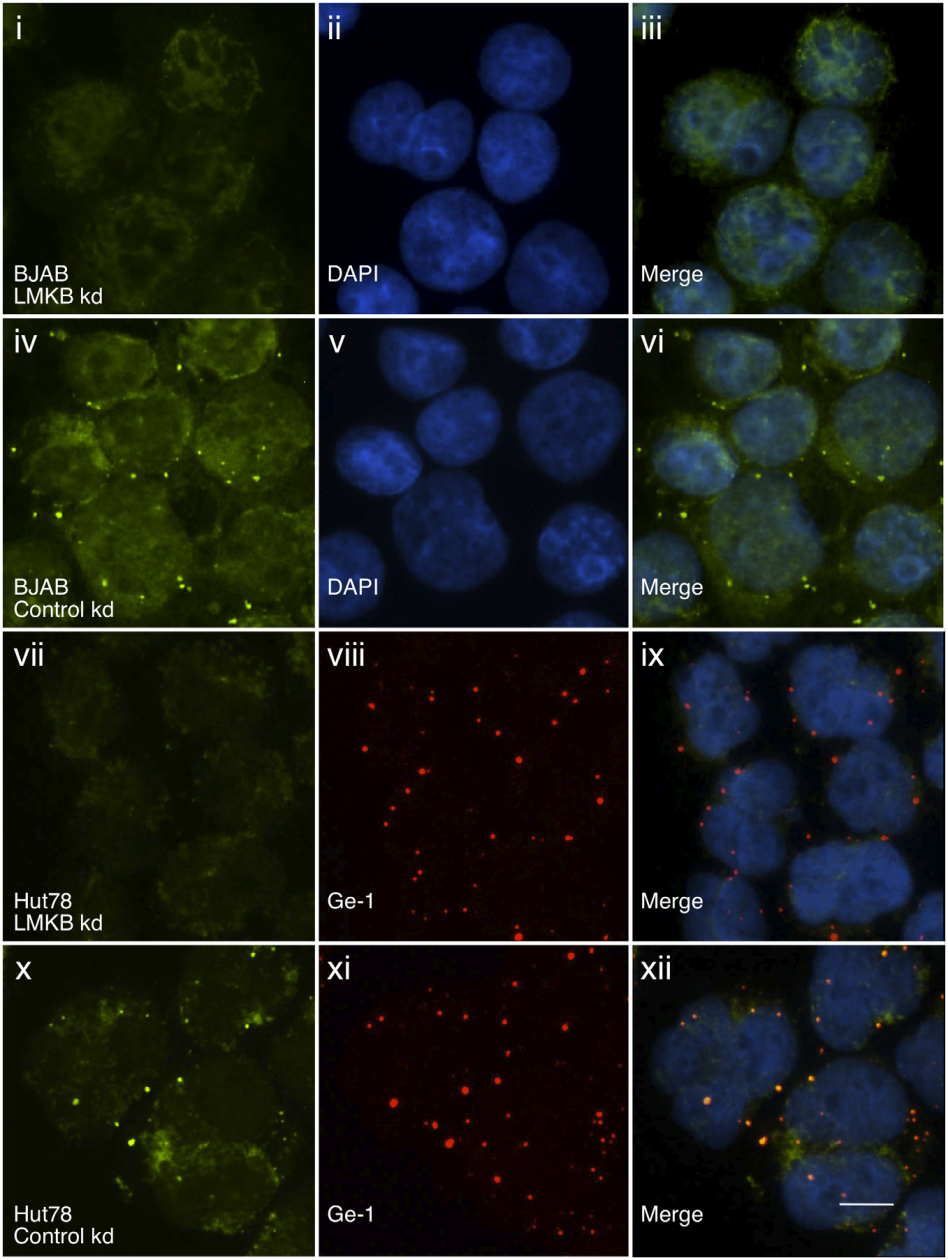
Loss of LMKB immunoreactivity after LMKB knockdown in BJAB and Hut78 cells. Treatment of BJAB cells with siRNA directed against LMKB resulted in loss of P-body staining as determined by indirect immunofluorescence (i). LMKB was detected in a cytoplasmic dot staining pattern in cells treated with control siRNA (green, iv). DAPI staining (blue) in ii and v indicate the location of nuclei. Merge of the preceding panels is shown in iii and vi. Treatment of Hut78 cells with siRNA directed against LMKB also resulted in loss of cytoplasmic dot staining (vii), compared with cells treated with control siRNA (green, x). Loss of LMKB did not alter the cellular location of Ge-1-containing P-bodies (red, viii and xi) demonstrating that LMKB is not required for P-body formation. Merge of vii and viii, and x and xi, is shown in ix and xii. DAPI staining (blue) in the merged panels indicates the location of nuclei. White bar in xii indicates 5.0 µm.

Su and colleagues showed that mutations in MARF1 resulted in upregulation of a cohort of transcripts, including *Line1* retrotransposon- and *PPP2cb*- mRNA [13]. To investigate whether depletion of LMKB in human T and B cell lines produced similar effects, we used siRNA to decrease *LMKB* mRNA and protein and measured *Line1* and *PPP2cb* mRNA levels. BJAB and Hut78 cells were treated with lentivirus particles containing LMKB or control siRNA. LMKB siRNA produced a greater than 80% decrease in *LMKB* mRNA levels in both BJAB and Hut78 cell lines (Figure 6, i). Immunoblot confirmed that the level of LMKB protein was markedly reduced in LMKB-depleted BJAB cells, compared with controls (Figure S2). In addition, after knockdown, LMKB was no longer detected by indirect immunofluorescence in either BJAB (Figure 7, i–vi) or Hut78 (Figure 7, vii) cells. Depletion of LMKB had no effect on staining for Ge-1 (Figure 7, vii-xii), suggesting that LMKB is not required for P-body integrity.

*Line1* mRNA was not detected by qRT-PCR in BJAB or Hut78 cells either before or after LMKB depletion. In addition, the level of *PPP2cb* mRNA was not affected by LMKB depletion in either cell line (Figure 6, ii). To further investigate the potential function of LMKB in human lymphocytes, we compared the LMKB-depleted BJAB transcriptome with that of control BJAB cells. Twelve genes, including *LMKB* itself, were down-regulated more than 1.8-fold. The mRNA levels of 15 genes were up-regulated more than 1.8-fold (Table 1). In BJAB cells, the microarray results indicated that there was a greater than five-fold increase in *IFI44L* mRNA, an increase that was confirmed using qRT-PCR (Figure 6, iii). An even greater increase in *IFI44L* mRNA was detected in LMKB-depleted Hut78 cells compared with control cells (LMKB knockdown vs control, 18 vs 1.0, p<.001). The mRNA level of other interferon inducible genes, such as *Mx-1*, was not altered by LMKB depletion.

**Table 1:** List of genes differentially expressed in BJAB cells after LMKB knockdown compared with control BJAB cells.

Gene	Affymetrix Gene ID	Fold Change	P-value
**Upregulated**			
IFI44L	7902541	5.34	1.91E-04
NCAM2	8067985	3.03	3.73E-03
AFF2	8170364	2.70	9.21E-03
HIST1H4D	8124413	2.36	1.43E-03
TTTY10	8177261	2.04	4.09E-02
LILRB1	8031223	1.96	6.69E-03
HIST1H2BM	8117594	1.96	4.46E-02
MME	8083494	1.92	3.34E-03
C6orf25	8178074	1.90	1.17E-02
MGC24125	8103906	1.88	8.72E-04
OLR1	7961142	1.86	1.04E-02
TUBA3E	8055194	1.85	1.38E-02
BACE2	8068671	1.82	6.31E-04
CHRNA6	8150550	1.81	6.88E-03
CXCL13	8095886	1.81	1.76E-02
**Downregulated**			
SPATS2L	8047272	−1.80	4.97E-04
JAG1	8064978	−1.81	1.96E-05
MKKS	8064967	−1.86	1.13E-04
DRP2	8168817	−1.89	7.07E-03
FGFR1OP2	7954492	−1.89	6.19E-05
ESF1	8065032	−1.94	2.51E-04
CRYBG3	8081171	−1.96	9.36E-03
PPP1R2P1	8125527	−2.07	3.65E-02
LOC644714	8161943	−2.07	4.07E-02
KIAA0430 (LMKB)	7999642	−2.26	1.99E-04
ANAPC13	8090866	−2.27	1.16E-05
MYBPC1	7957966	−3.03	1.45E-03

## Discussion

In this study, we used serum from a patient with autoantibodies directed against mRNA P-bodies to identify LMKB as a potential component of these structures. When expressed in HEp-2 cells together with FLAG-Ge-1, GFP-LMKB and FLAG-Ge-1 co-localized in P-bodies. Endogenous LMKB also localized to P-bodies, confirming that LMKB is a new P-body component. Depletion of LMKB in Hut78 cells did not alter the cellular distribution of Ge-1, suggesting that the presence of LMKB is not required for the integrity of P-bodies.

### Interaction between LMKB and Ge-1

A modified two-hybrid assay was used to test for interaction between LMKB and Ge-1. When GFP-LMKB was expressed in HEp-2 cells with Ge-1 fused to an exogenous NLS, both proteins localized to nuclear dots. When expressed in HEp-2 cells without NLS-Ge-1, GFP-LMKB localized in the cytoplasm. These results suggest that LMKB and Ge-1 interact inside the cell.

Expression of plasmids encoding fragments of LMKB together with NLS-Ge-1 revealed that the C-terminus of LMKB (amino acids 1622–1742) was the smallest portion of the protein capable of mediating interaction with Ge-1. The results of the two-hybrid assay were confirmed by studies, which showed that C-terminal portions of LMKB were able to co-precipitate FLAG-Ge-1 from COS-7 cell extracts.

To identify the portion of Ge-1 that mediates interaction with LMKB, plasmids encoding fragments of Ge-1 were co-expressed with full-length LMKB. Portions of Ge-1 that lack the C-terminus of Ge-1 (amino acids 935–1437) were unable to localize to P-bodies. However, when co-expressed with LMKB, Ge-1(1–1094) co-localized with LMKB in P-bodies. LMKB is the first protein identified to date that interacts with this portion of Ge-1.

### The Role of LMKB in Lymphocyte Cell Lines

Su and colleagues observed that homozygous mutation in LMKB, the murine orthologue of MARF1, resulted in defective oogenesis and impaired regulation of retrotransposon activity [13], [14]. Analysis of the MARF1 mutant oocyte transcriptome using microarrays revealed 377 transcripts that were increased and 27 decreased compared with wild-type oocytes. There was a marked up-regulation of *PPP2cb* mRNA, encoding the beta-isoform catalytic subunit of PP2A. Increased levels of PPP2cb may contribute to the observed meiotic arrest and infertility observed in *Marf1* ^−/−^ mice. Mutation in *MARF1* also increased the level of retrotransposon *Line1* mRNA in developing oocytes. Because retrotransposons may alter genomic stability by randomly inserting into the genome and inducing double-stranded DNA breaks, Su and colleagues concluded that murine LMKB might have a role in the maintenance of genomic integrity of developing oocytes [14].

In this study, we observed that LMKB was expressed in human T and B cell lines. Depletion of LMKB in BJAB and Hut78 cells did not produce the same effects as seen in LMKB-deficient oocytes. *Line1* retrotransposon mRNA was not detected in either control or LMKB-depleted cell lines. LMKB depletion also had no effect on the level of *PPP2cb* mRNA in lymphocyte cell lines. In contrast, depletion of LMKB in both Hut78 and BJAB cells increased the level of *IFI44L* mRNA. Although the function of *IFI44L* is unknown, increased *IFI44L* gene expression is a component of the Type 1 interferon response signature [32]. Increased levels of *IFI44L* mRNA are also part of the cellular response to viral infection [33]. The observation that LMKB may regulate the mRNA level of *IFI44L*, but not other components of the Type I interferon response signature, suggests that LMKB may have a specific, and perhaps subtle, role in limiting the inflammatory response.

In summary, in this study we identified LMKB, a putative RNA binding protein, as a component of mammalian mRNA P-bodies. The C-terminus of LMKB mediated interaction with Ge-1, while the N-terminus of Ge-1 was required for interaction with LMKB. Depletion of LMKB in T and B cell lines resulted in increased expression of *IFI44L*, a component of the Type I interferon response signature. In the absence of LMKB, *IFI44L* mRNA may fail to be recruited to mRNA P-bodies and may therefore retain a 5′cap and be resistant to 5′ ->3′ exoribonuclease-mediated degradation.

## Supporting Information

Figure S1 **LMKB co-localizes with DCP1 in mRNA processing bodies.** GFP-LMKB (green, i) localized to discrete, dot-like structures in the cytoplasm of transfected HEp-2 cells and co-localized with co-expressed FLAG-DCP1 (red, ii). Merge of fluorescence in i and ii is shown in iii. DAPI staining in iii (blue) indicates location of nuclei. White bar in iii indicates 5.0 µm.
(TIFF)

Figure S2 **LMKB knock-down decreased the level of immunoreactive LMKB in BJAB cells.** Immunoblot was used to further confirm that si-RNA-mediated knockdown decreased the level of LMKB protein in BJAB cells. Because of the relatively low level of LMKB in wild-type BJAB cells, human serum containing anti-LMKB antibodies was used to immunoprecipitate LMKB from BJAB cells prior to immunoblot. Precipitates were fractionated by SDS-PAGE, transferred to PVDF membranes and incubated with rabbit anti-LMKB antiserum. The level of LMKB was markedly reduced in LMKB-depleted (lane 3) compared with control (lane 1) BJAB cells. LMKB was not detected in either cell line when normal human serum (NHS) was used to immunoprecipitate LMKB (lanes 2 and 4).
(TIFF)
