# Supplementary figures and images for: LMKB/MARF1 Localizes to mRNA Processing Bodies, Interacts with Ge-1, and Regulates IFI44L Gene Expression
Source: PLoS One. 2014 Apr 22;9(4):e94784. doi: 10.1371/journal.pone.0094784 (PMC3995692; doi:10.1371/journal.pone.0094784)

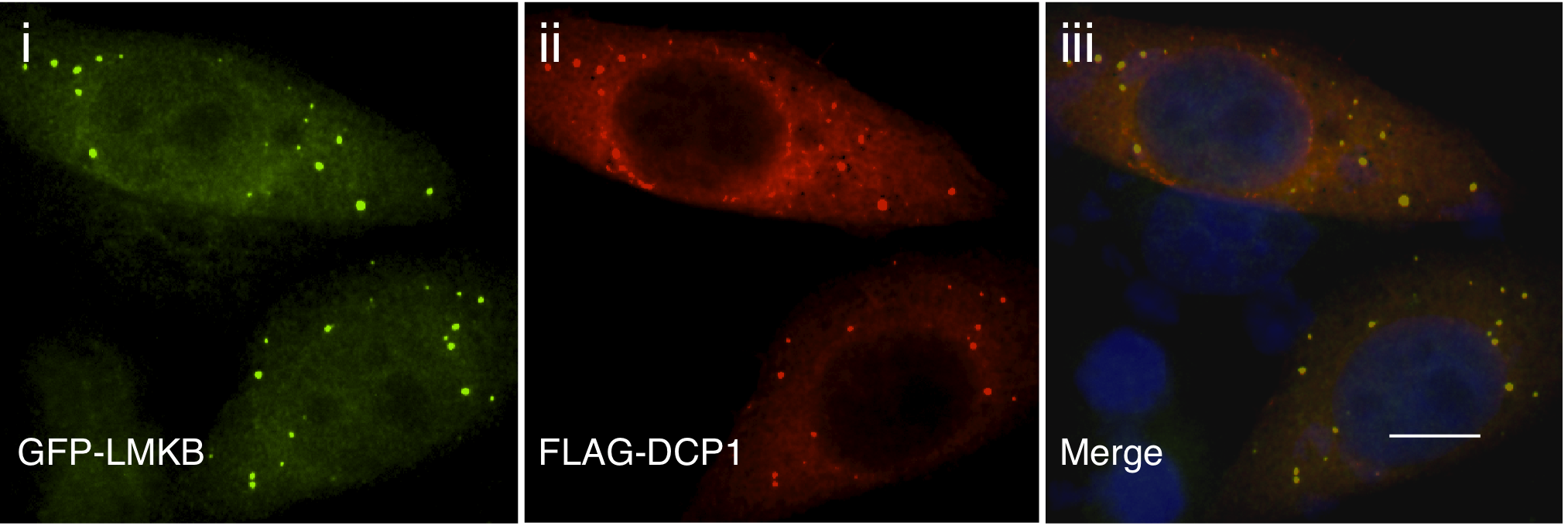

Supplement: Figure S1 — LMKB co-localizes with DCP1 in mRNA processing bodies. GFP-LMKB (green, i) localized to discrete, dot-like structures in the cytoplasm of transfected HEp-2 cells and co-localized with co-expressed FLAG-DCP1 (red, ii). Merge of fluorescence in i and ii is shown in iii. DAPI staining in iii (blue) indicates location of nuclei. White bar in iii indicates 5.0 µm. (TIFF) [file pone.0094784.s001.tif]

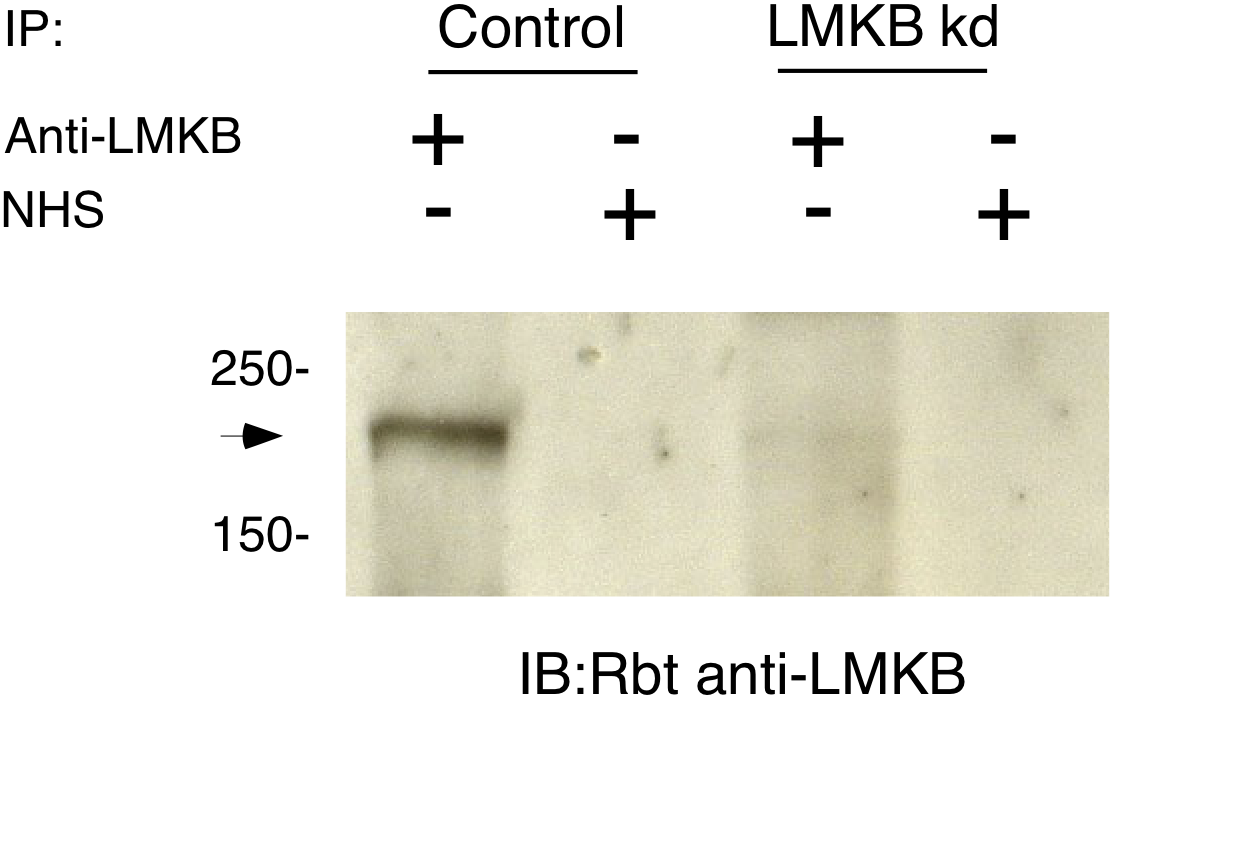

Supplement: Figure S2 — LMKB knock-down decreased the level of immunoreactive LMKB in BJAB cells. Immunoblot was used to further confirm that si-RNA-mediated knockdown decreased the level of LMKB protein in BJAB cells. Because of the relatively low level of LMKB in wild-type BJAB cells, human serum containing anti-LMKB antibodies was used to immunoprecipitate LMKB from BJAB cells prior to immunoblot. Precipitates were fractionated by SDS-PAGE, transferred to PVDF membranes and incubated with rabbit anti-LMKB antiserum. The level of LMKB was markedly reduced in LMKB-depleted (lane 3) compared with control (lane 1) BJAB cells. LMKB was not detected in either cell line when normal human serum (NHS) was used to immunoprecipitate LMKB (lanes 2 and 4). (TIFF) [file pone.0094784.s002.tif]
